# A cognitive dissonance-based mobile app intervention for smoking cessation: A single-arm pilot trial

**DOI:** 10.18332/tpc/221881

**Published:** 2026-08-07

**Authors:** Dana K. Smith, Herbert H. Severson, Vani N. Simmons

**Affiliations:** 1 Oregon Research InstituteSpringfieldUnited States; 2 Oregon Research Behavioral Interventions Strategies, Inc.SpringfieldUnited States; 3 Moffitt Cancer CenterTampaUnited States; 4 University of South FloridaTampaUnited States

**Keywords:** smoking cessation, cognitive dissonance, mobile app

## Abstract

**Introduction:**

Despite overall declines in the rate of tobacco use in the United States over the past 30 years, nearly 38 million Americans still smoke, posing extremely high risk for long-term health problems and death. Recent reviews note that there has been significant progress in the application of both clinical and public health interventions to help smokers quit; however, they have also concluded that the overall success rate has not significantly improved. There remains a need for novel, theory-driven approaches to enhance quit rates and innovative interventions that increase reach and engagement. In the current pilot study, we evaluated the use of a cognitive dissonance intervention for smoking cessation that was delivered via a mobile app.

**Methods:**

The *Support2Quit* app was evaluated in a single arm pilot study with 72 adult daily smokers who were assessed at baseline, 1 month and 3 months. Feasibility, usability, participant satisfaction, and intervention outcomes were evaluated.

**Results:**

The evaluation demonstrated promise that the *Support2Quit* app could effectively invoke significant increases in cognitive dissonance (average effect r=0.55; a large effect) and significant increases in readiness to quit smoking (average effect r=0.36; a medium effect). Outcomes showed a decrease in the number of cigarettes smoked each day for 41 (72%) of this sample; the non-parametric Wilcoxen signed-rank test showed an overall decrease in the number of cigarettes smoked per day from pretest to posttest (Z-score= -5.64, asymptotic p<0.001). User satisfaction ratings using a 5-point Likert scale indicated that the majority of 87.5% of participants reported liking the app (mean=3.7; SD=1.0) and that 82.5% reported that the app was helpful in reducing or stopping using cigarettes (mean=3.3; SD=1.0)

**Conclusions:**

Outcomes suggest that the *Support2Quit* mobile app was effective at inducing cognitive dissonance and might be a useful tool for reducing or quitting the use of cigarettes.

## Introduction

Despite overall declines in the rate of tobacco use in the United States over the past 30 years, improving quit rates among current smokers continues to be a significant public health focus given the high risk of long-term negative health outcomes^1^. There has been extensive research evaluating a wide range of smoking cessation approaches over the past 40 years^2,3^. Although there has been significant progress in the application of both clinical and public health interventions aimed at smoking cessation, researchers have concluded that the overall success rate of these interventions has not shown significant improvement^4-6^. This highlights the need for new, innovative, and theory-driven approaches to both improve the overall abstinence and long-term quit rates, as well as to increase the reach of effective interventions.

The utilization of mobile applications for smoking cessation and other addictive behaviors has increased over the past several years due to the low cost, ease of use, and increased availability via smartphones. While few research studies have reported on the effects of mobile behavioral-health-based interventions for smoking cessation, the results that are available have been promising^7^. Although NCI public health initiatives (e.g. QuitGuide, QuitSTART) are currently available, they have not been evaluated for efficacy.

One theory-driven approach that has shown promise for intervention development, but has yet to be examined when delivered via mobile app, is cognitive dissonance induction^8^. Cognitive dissonance has been widely studied and applied across physical and mental health fields, but has yet to be applied via a mobile app. Prior studies have shown that increasing cognitive dissonance, which is described as discomfort that one experiences when their behavior conflicts with their personal beliefs or values, has been shown to be efficacious in treating a variety of health behaviors, including treating obesity^9^, promoting healthy eating^10^, reducing fears^11,12^, managing chronic illness^13^, improving safe sex practices^14^, and preventing eating disorders^15^. Of particular relevance, cognitive dissonance approaches have also shown success in treating substance use^16,17^, reducing the initiation of smoking^18^, and improving short-term smoking cessation outcomes^19-21^. For instance, Simmons et al.^20^ found that smokers who engaged in video-taped discussions about the consequences of smoking (delivered via computer) had higher quit rates at follow-up at 1 month. Although these findings are promising, no significant long-term effects for cognitive dissonance activities for smoking cessation have been found, and the use of mobile apps for inducing cognitive dissonance has not been examined. Mobile app-based interventions offer the opportunity for a scalable approach to deliver repeated cognitive dissonance inducing activities over the course of treatment. The objective of this pilot study was to test the use of a mobile app that uses cognitive dissonance-inducing activities to aid in quitting smoking.

## Methods

### Study design

A prospective single-arm intervention study was conducted, using a pre/post-design. Participants were assessed at baseline, and at two points post-intervention, occurring at 1 and 3 months post-baseline.

### Participants and recruitment

Participants included 72 adult smokers who were recruited through social media in Lane County, Oregon. Inclusion criteria were: 1) aged ≥18 years; 2) self-reported daily smoking; 3) having a valid home mailing address in Lane County, Oregon; 4) English-speaking; 5) access to a smartphone with video capability for the duration of the project; 6) not currently enrolled in or participating in any tobacco cessation programs; and 7) an expressed desire to quit smoking.

Facebook advertisements were used to recruit current smokers interested in quitting smoking. Facebook users who clicked on the ad were directed to a Landing Page that provided information about the study and general eligibility criteria related to current smoking status. Interested individuals could then access a screening questionnaire to determine their eligibility for the study. Participants who were eligible for the study received additional study information and a link to an online consent form. The IRB-approved consent form described the purpose of the project, risks and benefits, eligibility criteria, and contact information for the study were provided. IRB approval for all study materials was received prior to recruitment.

### Study procedures

All participants in the pilot study were enrolled for a total of 3 months each and were assessed at baseline, and at 1 and 3 months post-baseline. Following the baseline assessment, participants received instructions and access information for downloading the *Support2Quit* app from the app store. Participants were assigned to groups of 8 using an automated process that occurred on a rolling basis as links to the app were distributed. The mobile app intervention was delivered over the course of one month. All consent and assessment procedures were conducted online using RedCap.

The pilot study evaluated feasibility, usability, cognitive dissonance, motivation to quit, and short-term outcomes such as making a quit attempt, use of the app (dosage), number of cigarettes smoked, and days abstinent from smoking at the assessment at 1 month. For the assessment at 3 months, quit attempts and cessation outcomes were assessed, including both point prevalence (i.e. no smoking in the past 7 days; no smoking in the past 30 days) and sustained abstinence, which is defined as self-reported abstinence in the assessments at 1 month and at 3 months.

Feasibility, usability, participant satisfaction, and intervention outcomes related to cigarette use were also evaluated. Data on: 1) frequency and duration of app use; 2) number of modules completed; 3) changes in measures of cognitive dissonance; and 4) changes in readiness to quit, and 4) changes in smoking attitudes and behaviors (including quit attempts, number of days without smoking, and co-use of other forms of tobacco, including e-cigarettes), were examined. Participants were compensated with a $25 gift card from Amazon for each of the assessments at baseline, 1 month, and 3 months. In addition, each participant who completed all three assessments received a bonus payment of $50.

### Intervention

Once the app was loaded onto their phone, participants in each group received access sequentially to the 6 intervention modules at the same pace, with one module released every 5 days over the course of a month. Each of the modules included an instructional video that described a cognitive dissonance inducing activity that participants were instructed to record and upload to the app for viewing by all group members. In addition to the 5 cognitive dissonance activities, participants received two smoking cessation tips per day designed to assist with reducing cigarette use and increasing motivation to quit, as well as to maintain participant engagement with the app on a daily basis.

#### Theory-based activities

The *Support2Quit* app was developed based on prior cognitive dissonance interventions. A common element of cognitive dissonance interventions is a focus on the intentional creation of dissonance to elicit behavior change. Regardless of the specific behavior being targeted, there are several common concepts that are central to effective cognitive dissonance induction. First, participation in the induction activity must be voluntary. Voluntary participation leads the individual to attribute the inconsistency between beliefs and behaviors to existing within themselves, rather than being due to the demands of a given situation^8^. Second, effortful involvement (i.e. actively engaging in treatment exercises) is also required, and is believed to result in greater dissonance and consequently greater motivation for change^22^. And finally, making public statements of beliefs (i.e. counter-attitudinal advocacy) is thought to elicit heightened dissonance responses^23^. In fact, Roehrig et al.^24^ have shown counter-attitudinal advocacy to be successful in isolation of the other dissonance-inducing components (i.e. voluntariness, effortful involvement). Counter-attitudinal advocacy activities that are often used to induce dissonance include preparing and delivering speeches or statements about personal beliefs, role-plays where individuals act out a particular behavior, or making public commitments. Given the central role that counter-attitudinal activities play in eliciting cognitive dissonance, these activities are a core component of the *Support2Quit* cognitive dissonance mobile app intervention.

The *Support2Quit* app incorporated each of these components of cognitive dissonance theory into 5 modules that were the basis of the intervention. The Introduction and the five cognitive inducing modules are as follows: 1) Introduction to *Support2Quit* and participant quit statement, 2) Costs – the costs of smoking and benefits of quitting, 3) Quit letter – commitment letter to family, 4) Pitfalls – quit plan; 5) Smoke free – plan for staying smoke-free; and 6) Letter to youth – advice to a youth on avoiding cigarette use. Each module involved at least one cognitive dissonance induction strategy. Module 1 required participants to make a voluntary statement of participation and intention to quit smoking. Modules 2–6 required participants to acknowledge incongruence between their attitudes, thoughts, and behaviors. Additionally, modules 3–6 required participants to make public expressions of counter-attitudinal viewpoints and to maximize their effort by requiring participants to create individualized counter-attitudinal statements to others that reflected their commitment to change. Each module included two videos: an instructional video that introduced the cognitive dissonance activity, and then a second video that was recorded by the participant and uploaded to the participant’s group within the app; uploaded videos were available for other group members to view and to provide encouraging comments. In addition to the 6 cognitive dissonance modules, participants received two tips per day; tips included such things as coping strategies, motivational messages, and reminders about the benefits of quitting. An example is: ‘Making your living environment smoke-free is an important part of your success. Remove all smoking reminders from your home, car, and workspace. Removing these reminders will help to free your mind’. Another example is: ‘Stay busy during your week of quitting. Make a list of as many healthy alternatives to smoking as you can think of and choose from this list when cravings hit’.

### Measures

Participants were assessed at three points: at baseline, 1 month (post-intervention), and at 3 months follow-up. All assessments were administered using RedCap. The baseline assessment collected information about participant risk factors, as well as past and current cigarette and other tobacco use, readiness to quit, quit attempts, and cessation in the past year. The assessment at 1 month evaluated app usability, cognitive dissonance, motivation to quit, and short-term outcomes such as making a quit attempt, app engagement (dosage), number of cigarettes smoked, and days abstinent from smoking. The assessment at 3 months was focused on cessation outcomes, including both point prevalence (i.e. no smoking in the past 7 days; no smoking in the past 30 days) and sustained abstinence, which was defined as sustained abstinence between the month 1 and month 3 follow-up assessments. Quit attempts were also collected at each assessment.

#### Demographics

Data on participants’ age, gender (male, female, non-binary, other), race/ethnicity, household income, education level, and health status were collected at baseline.

#### Nicotine dependence

Nicotine dependence was measured at baseline and at each follow-up assessment using the Fagerström test for nicotine dependence^25^ and items that assessed withdrawal experiences.

#### Past and current tobacco use

Past and current tobacco use was assessed at baseline and at each follow-up assessment via self-report, and included type (including e-cigarettes), frequency, and duration of use of all tobacco products.

#### Motivation to quit

Motivation to quit was assessed at baseline by asking participants if they wanted to quit within the next 30 days and at follow-up assessments in terms of stages of change^26^. In addition, an adaptation of the Contemplation Ladder^27^ that we have used extensively in our prior research^28,29^ and have found that predicting tobacco cessation was also administered^28,30^.

#### Quit attempts

Quit attempts were measured via self-report, indicated by the number of intentional quit attempts that lasted at least 24 hours, duration of quitting, and use of pharmacological adjuncts at each time point.

#### Cognitive dissonance

Cognitive dissonance was measured using the Dissonance Thermometer^31,32^. Prior research has shown that the 3-item discomfort factor of the Dissonance Thermometer represents the affective expression of cognitive dissonance^32^. Cognitive dissonance was assessed after each cognitive dissonance activity, as well as at each of the assessments at baseline, month 1, and month 3.

#### Usability

Program navigation and usability were assessed using the System Usability Scale. In addition, participants were asked to report on: 1) ease of use, 2) perceived benefits of using the app, and 3) suggestions for app modifications. In addition, participants provided ratings on satisfaction with the app and usability on a 7-point Likert scale.

### Analytic goals and plan

The evaluation had four main goals: 1) determine the amount of *Support2Quit* app use; 2) determine whether each activity in the app could increase cognitive dissonance and readiness to quit smoking relative to pretest levels; 3) determine whether change in readiness to quit smoking was related to changes in smoking; and 4) determine the participant’s usability and satisfaction to the *Support2Quit* app.

A descriptive summary of the program use data, was used to summarize and evaluate the amount of *Support2Quit* app use. Paired t-tests were used to determine whether or not there were significant increases in cognitive dissonance and readiness to quit smoking from pre- to post-intervention in relation to dosage (number of modules completed). The point-biserial r is provided as a measure of effect sizes with the convention: 0.14 small, 0.36 medium, and 0.51 large^33^. The non-parametric Wilcoxon signed-rank test was used to evaluate whether participants were smoking fewer cigarettes per day, and Pearson correlations, with the convention 0.10 small, 0.30 medium, and 0.50 large^34^, evaluated the extent to which readiness to quit smoking was associated with change in smoking from the pretest to posttest. Examination of descriptive statistics for the user satisfaction items informed participants' reaction to the program.

Due to conceptual concerns about imputing smoking status, decreases in smoking from pretest to posttest were evaluated with participants who completed both the pretest and posttest assessment (n=54). Although a decrease in power was realized, as demonstrated above, failure to provide posttest data was not significantly associated with any baseline smoking characteristics or study outcomes, indicating a low likelihood of bias when interpreting observed data only. Also, the number of participants who provided cognitive dissonance and readiness to quit data after downloading the app content was a function of app use and resulted in data from only 17 to 25 participants. A *post-hoc* power analysis showed the study was only powered to detect significant correlations between change in cigarettes smoked per day and readiness to quit scores of r=0.51–0.62 or greater. Thus, to evaluate the relationships between readiness to quit smoking and observed decreases in the number of cigarettes smoked, we rely on the effect size convention for correlations of 0.2 small, 0.5 medium, and 0.8 large effects^34^, rather than statistical significance.

An exploratory complier analysis compared participants who downloaded and watched all the content to those who did not, examining abstinence rates at posttest. Logistic regression models, estimated with a logit link and reporting of odds ratios and 95% confidence intervals, were used to compare the posttest abstinence rates by complier status.

## Results

### Participants

A total of 72 participants completed the pretest assessment, and 54 (75%) completed the posttest assessment. Participants who completed both assessments were compared to those who did not on all demographic and baseline smoking characteristics. No significant differences were found with the exception of age; participants who dropped out of the study were on average 43.9 years at pretest compared to 49.9 for participants who completed both assessments. The 72 participants in the study were mostly female (76%) and on average aged 48.4 years (SD=11.0). Table 1 provides complete data for all demographic characteristics and baseline smoking rates.

**Table 1:** Baseline study demographics (N=72)

Characteristics	n	%
**Race**		
American Indian/Alaskan Native	3	4.2
Asian	1	1.4
African American/Black	1	1.4
White	59	81.9
Other	7	9.7
Multiple races	1	1.4
**Number of children in the home**		
2	9	18.0
3	15	30.0
4	17	34.0
≥5	9	18.0
**Marital status**		
Single	13	18.1
Married/living together as married	36	50.0
Partnered/not living together	3	4.2
Separated	3	4.2
Divorced	12	16.7
Widowed	5	6.9
**Education level**		
Did not graduate high school	2	2.8
High school graduate	10	13.9
Some college, no degree	26	36.1
Technical school or Associate’s degree	16	22.2
Bachelor’s degree	11	15.3
Master’s degree	6	8.3
Doctorate degree	1	1.4
**Household income ($)**		
<10000	3	4.3
10000–19999	4	5.7
20000–29999	14	20.0
30000–39999	11	15.7
40000–49999	7	10.0
50000–59000	9	12.9
60000–69000	5	7.1
70000–79000	4	5.7
80000–89000	4	5.7
≥90000	9	12.9
**Duration smoked daily (years)**		
≤1	1	1.5
1–3	4	5.5
4–9	9	12.5
≥10	58	80.5
**Cigarettes smoked daily**		
≤5	1	1.4
6–10	18	25
11–15	15	20.8
16–20	24	33.3
21–30	11	15.3
≥31	3	4.2

### Increases in cognitive dissonance and readiness to quit smoking

Table 2 shows the pretest and post-content scores for cognitive dissonance and readiness to quit smoking. Results show that there were significant increases in cognitive dissonance measured immediately following each of the intervention modules. Four of the five modules were associated with large effects: ‘Costs’ (p=0.003), ‘Quit letter’ (p<0.001), ‘Pitfalls’ (p=0.004), and ‘Letter to youth’ (p=0.042). The only module that was not associated with significant increases in cognitive dissonance was ‘Staying smoke-free’ (p=0.098). Although only one statistically significant increase was detected for readiness to quit smoking (‘Pitfalls’: p=0.043), all readiness to quit scores increased from pre- to posttest, and with the exception of the readiness to quit smoking score associated with the ‘Costs’ module, the increases were all medium or medium to large effect sizes. Taken together, these results show that the *Support2Quit* app could invoke significant increases in cognitive dissonance in response to each of the five intervention modules (average effect r=0.55; a large effect) and meaningful increases in readiness to quit smoking (average effect r=0.36; a medium effect).

**Table 2:** Results of change in cognitive dissonance and readiness to quit smoking at 1 month

	Total n	Level of cognitive dissonance				Test statistics		
		Pretest		1 Month				
		Mean	SD	Mean	SD	t	p	r
**Watched and downloaded app content and corresponding cognitive dissonance scores**								
Costs	25	2.23	1.23	3.32	1.43	3.27	0.003	0.56
Quit letter	21	2.27	1.24	4.33	1.48	4.93	<0.001	0.74
Pitfalls	21	2.38	1.34	3.67	1.76	3.29	0.004	0.59
Smoke-free	18	2.35	1.36	3.14	1.83	1.75	0.098	0.39
Letter to youth	17	2.33	1.38	3.69	1.85	2.12	0.042	0.49
**Watched and downloaded app content and corresponding readiness to quit scores**								
Costs	25	7.52	1.63	7.64	2.20	0.29	0.776	0.06
Quit letter	21	7.67	1.83	8.24	1.81	1.29	0.219	0.28
Pitfalls	21	7.61	1.54	8.81	1.54	2.16	0.043	0.43
Smoke-free	18	7.67	1.75	8.50	1.86	1.28	0.219	0.30
Letter to youth	17	8.00	1.60	8.93	1.75	1.71	0.110	0.42

### Decrease in smoking and relationship to readiness to quit smoking

Table 3 shows the number of cigarettes smoked on a typical day during the study. From pretest to posttest, 41 (72%) participants reported lower daily smoking rates at posttest, one participant (2%) reported a higher rate, and 12 (26%) participants reported the same rates. The non-parametric Wilcoxon signed-rank test showed the overall decrease in number of cigarettes smoked per day was significantly lower at posttest (*Z*-score= -5.64, asymptotic p<0.001).

**Table 3:** Number of cigarettes smoked per day

	Baseline		1 Month	
**How many cigarettes per day do you usually smoke?**	n	%	n	%
≤5	0	0.0	13	28.3
6–10	14	25.9	16	34.8
11–15	15	27.8	5	10.9
16–20	13	24.1	9	19.6
21–30	12	22.2	3	6.5
≥31	0	0.0	0	0.0

A daily smoking change score was computed by subtracting the posttest number of cigarettes smoked per day rating from the pretest rating. Thus, a lower score indicates less daily smoking at posttest. The smoking change score was correlated with the readiness to quit smoking scores reported after downloading the *Support2Quit* content. All correlations between change in number of cigarettes smoked and readiness to quit scores were negative, indicating that the greater the readiness to quit smoking, the fewer cigarettes smoked at posttest. Although only one statistically significant correlation was detected (p=0.037), the average correlation among the five readiness to quit smoking scores and change in number of cigarettes smoked was r= -0.38, a medium effect. In order from smallest to largest, the correlations between change in number of cigarettes smoke and readiness to quit smoking after watching and downloading the ‘Costs’ content (r= -0.10; small effect), ‘Quit letter’ content (r= -0.26; small effect), ‘Pitfalls’ content (r= -0.43; medium effect), ‘Smoke-free’ content (r= -0.58; medium effect), and ‘Letter to youth’ content (r= -0.54; medium effect).

### App usability and satisfaction

Of the participants who used the app, 40 provided feedback on the *Support2Quit* app usability and satisfaction. A descriptive summary of the feedback is summarized in Table 4 and includes the percentage that endorsed each category (on a Likert scale 1–5), and the mean and standard deviation for each item. Overall, 87.5% reported liking the app (mean=3.7; SD=1.0), and 82.5% reported that the app helped to reduce their cigarette use (mean=3.3; SD=1.0). Additionally, 75% of participants reported that the *Support2Quit* app was easy or very easy to use (mean=4.3; SD=0.9), and 65% agreed or strongly agreed they would recommend the *Support2Quit* app to a friend (mean=3.7; SD=1.3).

**Table 4:** Proportion (%) of participant ratings on a 1–5 Likert scale of Support2Quit app usability and satisfaction at 3 months

1	2	3	4	5	Mean	SD
**Overall**						
Disliked a lot	Disliked	Somewhat liked	Liked	Liked a lot		
2.5	10.0	25.0	37.5	25.0	3.7	1.0
**How easy was it to use?**						
Very hard	Hard	Somewhat easy	Easy	Very easy		
0.0	7.5	7.5	35.0	50.0	4.3	0.9
**How well did it address issues you have about quitting smoking?**						
Not at all	A little bit	Somewhat	Quite a bit	A lot		
5.0	12.5	35.0	32.5	15.0	3.4	1.1
**How much new information did it provide?**						
Not at all	A little bit	Somewhat	Quite a bit	A lot		
15.0	15.0	42.5	15.0	12.5	3.0	1.2
**How would you rate it in helping you reduce or stop using cigarettes?**						
Very unhelpful	Unhelpful	Somewhat helpful	Helpful	Very Helpful		
5.0	12.5	40.0	30.0	12.5	3.3	1.0
**How helpful were the activities that you did, such as writing letters and posting videos?**						
Very unhelpful	Unhelpful	Somewhat helpful	Helpful	Very Helpful		
10.0	7.5	40.0	30.0	12.5	3.3	1.1
**How helpful was it to watch the videos posted by your group members?**						
Very unhelpful	Unhelpful	Somewhat helpful	Helpful	Very Helpful		
5.1	7.7	28.2	48.7	10.3	3.5	1.0
**How helpful were the daily supportive messages from the app?**						
Very unhelpful	Unhelpful	Somewhat helpful	Helpful	Very Helpful		
2.5	7.5	37.5	32.5	20.0	3.6	1.0
**I would use it frequently if I was needing support to avoid using cigarettes**						
Strongly disagree	Disagree	Neither agree nor disagree	Agree	Strongly agree		
7.5	12.5	15.0	32.5	32.5	3.7	1.3
**It is available when I need it**						
Strongly disagree	Disagree	Neither agree nor disagree	Agree	Strongly agree		
2.5	2.5	15.0	32.5	47.5	4.2	1.0
**It motivates me to do well**						
Strongly disagree	Disagree	Neither agree nor disagree	Agree	Strongly agree		
10.0	5.0	25.0	30.0	30.0	3.7	1.3
**I thought it was unnecessarily complex**						
Strongly disagree	Disagree	Neither agree nor disagree	Agree	Strongly agree		
28.3	53.8	12.8	2.6	2.6	2.0	0.9
**I would recommend it to my friends**						
Strongly disagree	Disagree	Neither agree nor disagree	Agree	Strongly agree		
10.0	7.5	17.5	32.5	32.5	3.7	1.3

## Discussion

This pilot evaluation of the *Support2Quit* app demonstrated a decrease in the number of cigarettes smoked each day for 72% of this sample of long-term, moderate to highly dependent smokers, with 80.5% having smoked for ≥10 years. While cessation of smoking is the goal, a reduction in smoking can have significant health benefits and make it easier to quit^6^. The evaluation also demonstrated promise that the *Support2Quit* app could invoke significant increases in cognitive dissonance in response to the five intervention modules, as well as increases in readiness to quit smoking. There was also evidence to suggest that increases in the readiness to quit smoking scores were related to decreases in daily smoking of cigarettes from pretest to posttest. Although compliance with using the app was less than ideal for this moderately to highly nicotine-dependent sample, those who did use the *Support2Quit* app indicated high usability and satisfaction ratings. For example, 75% of participants found the *Support2Quit* app easy or very easy to use, and 65% agreed or strongly agreed they would recommend the *Support2Quit* app to a friend. Increasing engagement should be a goal for future use of the mobile app since higher participation and completion of cognitive dissonance activities appear to be associated with higher quit rates. Possible ways to increase engagement and completion of videos by participants might include resending the request for the activity with a narrator providing an example for them to emulate, and proactive texts to the participant about the benefits of sharing videos with other group members, as well as prompts to participants to view other videos uploaded by group members. Additionally, tailored notifications providing encouragement that match the participant’s progress in the app (e.g. ‘Congrats on finishing module 2! Your next module is available now.’).

Even more encouraging are the benefits of the *Support2Quit* app for the participants who completed all of the study content. For those participants who completed all of the content (n=17), 29% reported 1 week abstinence from smoking cigarettes at posttest compared to only 8% who did not watch and download all the content. This difference in 7 day point prevalence abstinence was associated with an odds ratio of 4.72, indicating those who watched and downloaded all the *Support2Quit* content had 372% greater odds of abstinence from smoking in the past week at posttest compared to those who did not watch and download all content, a near-significant difference. Given the excessively wide confidence interval, this finding should be interpreted cautiously and replicated in future studies.

### Limitations

This study has several limitations. First, this pilot study did not include a control group for comparison, which limits the ability to determine whether the observed changes in smoking were due to the *Support2Quit* app or to other external variables. Lack of a control group also limits the ability to determine the significance of the observed changes. Second, this pilot included a small sample size that was selected through voluntary participation, which could lead to selection bias and also limits the ability to generalize findings to a larger population. Additionally, the small sample size resulted in limitations to the analyses, as the study was not statistically powered to effectively detect intricacies in the relationships between use of the app, readiness to quit, and outcome variables, or to examine differences related to age, gender, and race/ethnicity. Additionally, reliance on paired t-tests and multiple correlation analyses can increase the risk of Type I error. This study relied on self-report data and provided incentives for participation, which presents potential issues related to social desirability and recall bias, and could also affect engagement.

## Conclusions

This pilot examination of the *Support2Quit* app provides encouraging preliminary outcomes for smoking cessation, as well as for the ability to elicit cognitive dissonance with the use of a mobile app. Additionally, usability data suggest that users of the *Support2Quit* app found the app engaging and easy to use. A fully powered clinical trial is needed to assess the efficacy of the *Support2Quit* app for smoking cessation.
